# Resilience to aging in the regeneration‐capable flatworm *Macrostomum lignano*

**DOI:** 10.1111/acel.12739

**Published:** 2018-02-28

**Authors:** Stijn Mouton, Magda Grudniewska, Lisa Glazenburg, Victor Guryev, Eugene Berezikov

**Affiliations:** ^1^ European Research Institute for the Biology of Ageing University of Groningen University Medical Center Groningen Groningen The Netherlands

**Keywords:** aging, flatworms, gene expression, regeneration, rejuvenation, stem cells

## Abstract

Animals show a large variability of lifespan, ranging from short‐lived as *Caenorhabditis elegans* to immortal as *Hydra*. A fascinating case is flatworms, in which reversal of aging by regeneration is proposed, yet conclusive evidence for this rejuvenation‐by‐regeneration hypothesis is lacking. We tested this hypothesis by inducing regeneration in the sexual free‐living flatworm *Macrostomum lignano*. We studied survival, fertility, morphology, and gene expression as a function of age. Here, we report that after regeneration, genes expressed in the germline are upregulated at all ages, but no signs of rejuvenation are observed. Instead, the animal appears to be substantially longer lived than previously appreciated, and genes expressed in stem cells are upregulated with age, while germline genes are downregulated. Remarkably, several genes with known beneficial effects on lifespan when overexpressed in mice and *C. elegans* are naturally upregulated with age in *M. lignano*, suggesting that molecular mechanism for offsetting negative consequences of aging has evolved in this animal. We therefore propose that *M. lignano* represents a novel powerful model for molecular studies of aging attenuation, and the identified aging gene expression patterns provide a valuable resource for further exploration of anti‐aging strategies.

## 1 INTRODUCTION

During the last decade, the large diversity of age‐related changes and life trajectories of different animals became increasingly clear (Jones et al., 2014). Studying species with aging profiles different from those of the established aging models, such as *Caenorhabditis elegans*,* Drosophila melanogaster*, and mice, offers insight into naturally occurring mechanisms to delay or suppress senescence and age‐related phenotypes. Particularly interesting are the few animals which are claimed to be immortal, such as *Hydra*, for which compelling evidence of a nonsenescent life trajectory exists (Martínez, 1998; Schaible et al., 2015) and certain flatworms (Haranghy & Balázs, 1964; Lange, 1968; Tan et al., 2012).

Already at the start of the 20th century, it was suggested that flatworms age, but are also able to reverse the aging process (rejuvenate) through regeneration of the body after amputation or fission (Child, 1915). As a consequence, repeated rejuvenation can potentially lead to immortality. While the original experiments by Child (Child, 1915) and Hyman (Hyman, 1919) were debated due to contradictory results (Allen, 1919; Brøndsted, 1969; Pedersen, 1956), several observations of an extended lifespan after regeneration further popularized the rejuvenation hypothesis (Egger, Ladurner, Nimeth, Gschwentner & Rieger, 2006; Haranghy & Balázs, 1964). Poorly defined culture conditions and potential non‐age‐related causes of mortality might, however, create some doubt about these findings. In addition, one should keep in mind that a lifespan extension does not necessarily include a reversal of aging as it might also be the result of a decelerated or temporally arrested aging (Hass, 2009). A more recent study focusing on telomere shortening and elongation in *Schmidtea mediterranea* indicated an additional point of discussion, as it demonstrated that only the asexual strain, and not the sexual strain, is able to reverse age‐dependent changes in telomere length after regeneration (Tan et al., 2012). In conclusion, unambiguous proof of rejuvenation on the organismal level after regeneration is still absent, and more research is required to understand flatworm aging and how it is affected by (multiple) regeneration.

This study focuses on the sexual flatworm species *Macrostomum lignano* (Rhabditophora), which is a non‐self‐fertilizing hermaphrodite (Ladurner et al., 2008). The worms have a large mesodermal population of proliferating cells, called neoblasts, which can form every cell type in the body (Ladurner et al., 2008). These neoblasts enable a high cellular turnover during homeostasis and a remarkable regeneration capacity (Egger et al., 2006; Ladurner et al., 2008). *Macrostomum* is able to regenerate missing body parts anteriorly, posteriorly, and laterally, although the presence of the brain and pharynx is obligatory. Interestingly, somatic neoblasts are able to regenerate functional gonads, indicating the lack of a strict separation between soma and germline (Egger et al., 2006). Previously, it was shown that *M. lignano* ages (Mouton, Willems, Back, Braeckman & Borgonie, 2009) and that repeated amputations might extend the lifespan (Egger et al., 2006).

Here, we tested the rejuvenation hypothesis in *M. lignano* by characterizing survival, morphology, fertility, and gene expression as a function of age in intact, and single and multiple regenerated worms. We found that regeneration does not affect the aging *of M. lignano*, but that this worm evolved mechanisms to attenuate its aging process. The generated temporal gene expression profile of *M. lignano* provides an insight into these mechanisms and will serve as a valuable resource for aging research.

## 2 RESULTS

### 2.1 Regeneration does not extend lifespan in *Macrostomum lignano*

**Figure 1:**
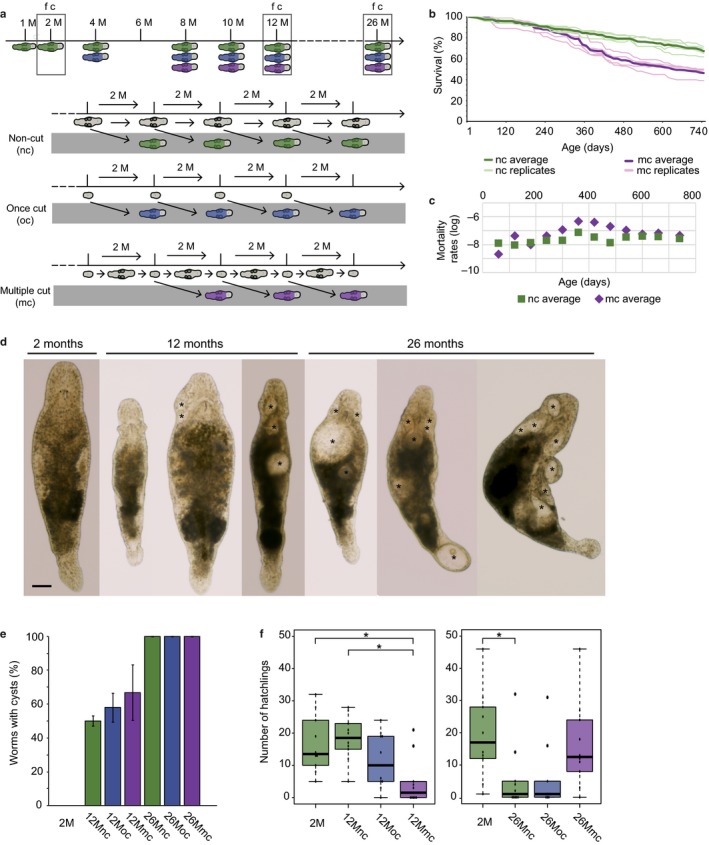
Experimental design, survival, morphology, and age‐related fertility of *Macrostomum lignano*. (a) Schematic representation of the experimental setup. RNA of worms was collected at all indicated ages (M = months). In addition, fertility (**f**) and the number of worms with cysts (**c**) were quantified at the age of 2, 12, and 26 months. The study included three conditions: intact (NC, green), once cut (OC, blue), and multiple cut (MC, purple) animals. How worms are obtained for each condition is visualized below the timeline. The gray area represents the animals collected for studies, while the animals above represent the aging culture. (b) Survival of intact and multiple cut *Macrostomum lignano* DV1 animals. For both conditions, the five separate replicates, each starting with 100 individuals, and the average survival curve in bold are visualized. (c) Average mortality rates of intact and multiple cut worms. Note the temporal increase at the age of about a year for multiple cut worms. (d) Morphology of *Macrostomum lignano* as a function of age. Young intact worm without morphological abnormalities (2 months) and aged intact worms (12 and 26 months) are shown. Most noticeable are the lack of eyes and the presence of cysts (indicated with asterisk), which vary in size, location, and number per worm. Scale bar 100 μm. (e) Percentage of worms with cysts as a function of age. (f) Fertility of the three conditions at the age of 12 and 26 months. For both ages, a control of young 2‐month‐old worms is included (*p < .05)

To test the effect of single and multiple regenerations on the aging process of *M. lignano*, we studied several characteristics such as survival, morphology, fertility, and gene expression in intact animals (noncut, NC), worms which regenerated their body once (once cut, OC), and animals which regenerated their body multiple times (multiple cut, MC). Regeneration was induced by amputating the head above the testes. We observed that the regenerated animals started to produce progeny around 1 month after amputation, indicating that the reproductive function is fully restored by that time. Therefore, to ensure that all animals in the group are fully regenerated, we provided extra time for regeneration and chose two‐month intervals between consecutive amputations for the MC condition. A general design of the complete experimental setup is visualized in Figure 1a. The three regenerative conditions were studied simultaneously, but several aging cultures were started at different time points to account for potential culture condition biases (Table S1).

Multiple amputations of the head and subsequent regeneration led to a decreased survival compared to intact worms. An average survival of 50% (median lifespan) was reached at day 620 for the MC worms, while the NC worms had an average survival of 73% (Figure 1b and Table S2). This difference is, however, caused by a temporal increased mortality rate at the age of about 1 year, which is more pronounced in the MC worms compared to the NC worms. After reaching 540 days, the chance of dying was not significantly different between both conditions (Figure 1c). Interestingly, we did not observe a decreasing regenerative capacity with advancing age or with an increasing amount of amputations. At all ages, worms regrew a complete body in about 3 weeks.

With advancing age, specific morphological changes were observed, such as the loss of eyes and the presence of cysts, which are internal body cavities (Figure 1d). The size, number, and location of observed cysts varied greatly. However, we did not observe negative effects of cyst formation on the survival or regeneration capacity of worms. Only in rare cases of very large cysts or a very large number of them, an indirect negative effect was observed, for example, by the inhibition of movement. The percentage of worms having cysts was compared between intact young controls (2M) and NC, OC, and MC worms at the age of 12 and 26 months. While the percentage of worms having cysts significantly increased with age (*p* ≤ .001, ANOVA with post hoc Bonferroni test), there were no significant differences within each age group (Figure 1e). As cysts often appear in the head, regeneration of the body did not have a large impact on these numbers. While regeneration would initially result in cyst‐free body, cysts reappeared after regeneration in aged worms.

At the age of 26 months, NC worms produced significantly less offspring than 2‐month‐old worms (*p* < .05, Kruskal–Wallis Test with post hoc Dunn's multiple comparison tests), suggesting that fertility decreases with advancing age (Figure 1f). Interpreting the effect of regeneration of the body and gonads on fertility is more difficult. At the age of 12 months, the MC worms produced significantly less juveniles than NC worms of the same age and young controls (*p* < .05, Kruskal–Wallis Test with post hoc Dunn's multiple comparison tests). This reduced fertility of MC worms compared to young controls was, however, not observed at the age of 26 months (Figure 1f).

Taken together, the generated survival, morphology, and fertility data demonstrate that single and multiple amputations and consequent regeneration do not cause a slowing down or reversal of aging in *M. lignano*.

### 2.2 The long‐lasting effect of regeneration at the transcriptional level is limited

**Figure 2:**
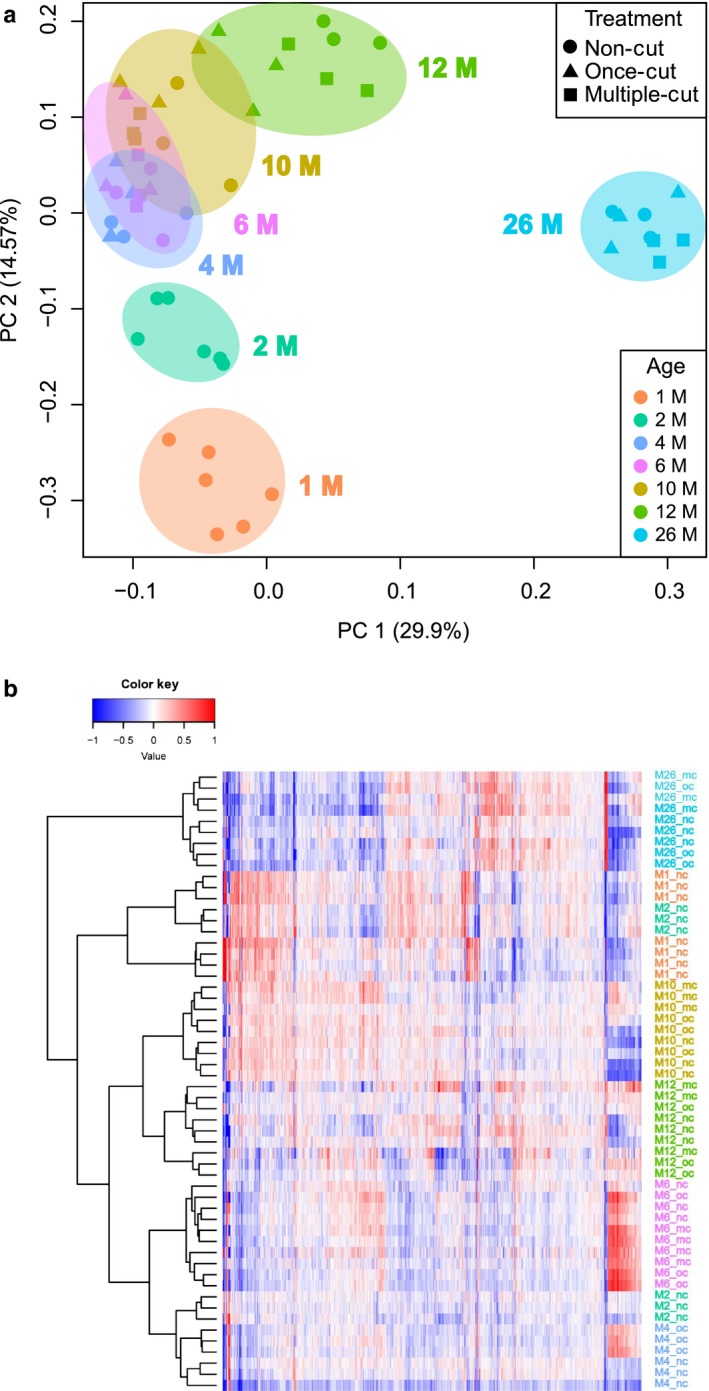
Clustering of treatment and age groups of *Macrostomum lignano* based on gene expression. (a) Principal component analysis based on the top 1,000 most variable genes in the dataset. Samples are clustered primarily by age and not by treatment. (b) Heatmap and hierarchical clustering of samples based on the same 1,000 most variable genes as in (a)

To investigate how regeneration affects aging at the transcriptional level, we generated in total 54 RNA‐seq libraries in triplicates for all treatment conditions at 1, 2, 4, 6, 10, 12, and 26 months (Table S1). Principal component analysis revealed that single and multiple regenerations do not dramatically change expression profiles, and instead, the age of the samples is the primary source of gene expression variance, with the first principal component explaining 29.9% of the variance and separating advanced age samples (12M and 26M) from the younger age samples, and the second principal component explaining 14.6% of the variance and separating 1M, 2M, 4M and 6M, and 10M samples (Figure 2a). Hierarchical clustering of the samples also revealed grouping of the samples mainly by age and indicated the absence of a rejuvenation effect after regeneration (Figure 2b). Thus, the gene expression data confirm our phenotypic observation that regeneration has no significant effects on the age of *M. lignano*. Furthermore, the analysis revealed that the 1M samples are substantially different from the 2M samples, most likely because the animals are still growing at this stage. For this reason, we chose 2M samples as a young adult reference for the subsequent analysis.

**Figure 3:**
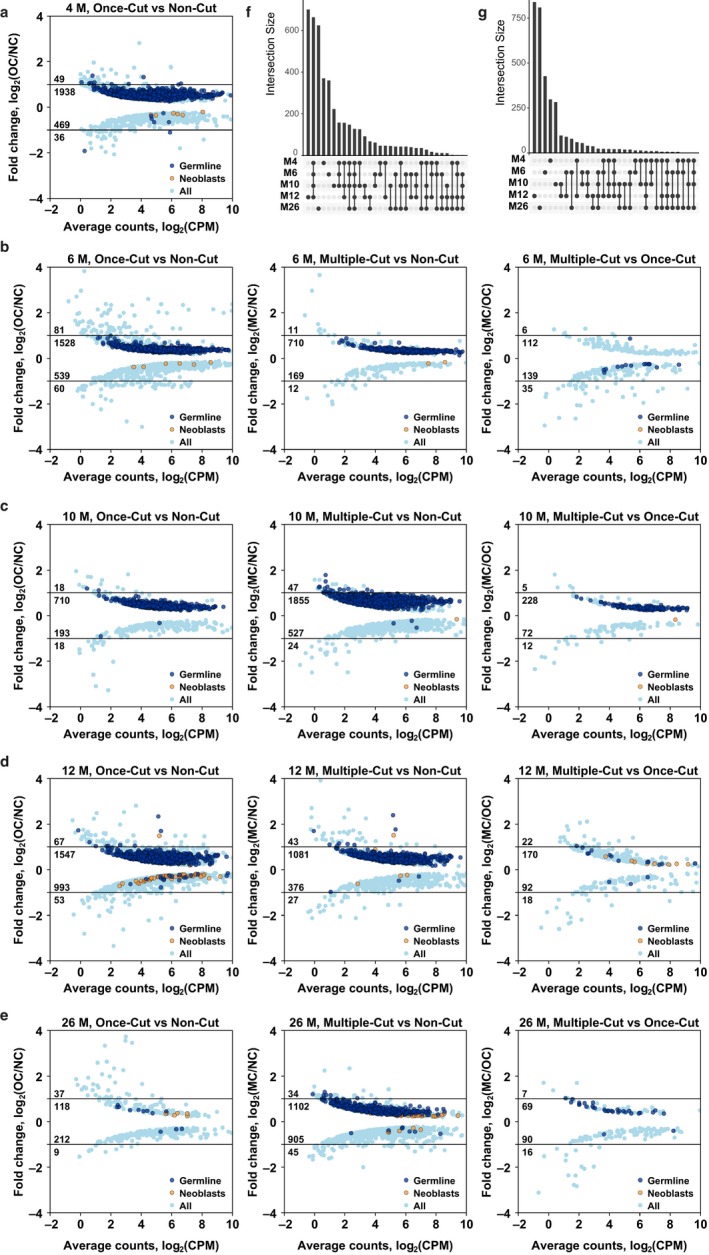
Differentially expressed genes after single and multiple regenerations in different age groups. (a–e) 4M, 6M, 10M, 12M, and 26M time points, respectively. (f, g) Overlap between genes upregulated (f) and downregulated (g) after regeneration in different age groups

We next focused on investigating gene expression changes between regenerated and control animals within age groups. Two months after amputation, differentially expressed genes could still be found in each of the studied age groups (Figure 3a–e). The vast majority of them, however, had a fold change <2. Comparison of MC and OC worms resulted in much less differentially expressed genes than the comparison of one of those conditions with NC worms of the same age (Figure 3b–e), which further indicates that, for at least up to 12 amputations, the number of regeneration events had little long‐lasting effects. Overlapping up‐ and downregulated genes at different ages showed that a substantial fraction of upregulated genes is shared between different ages (Figure 3f), whereas downregulated genes are mostly age‐specific (Figure 3g). We then investigated the distribution of the previously described “neoblast” and “germline” gene groups (Grudniewska et al., 2016) among the identified differentially expressed genes. Strikingly, genes with significantly increased expression 2 months after single or multiple amputation of the body appear to be fivefold to 10‐fold enriched (*p* < .001 in all comparisons) in proliferating germline cells (Figure 3a–e and Table S3), indicating that the effects of gonad regeneration on the gene expression level last much longer than expected based on morphology. To investigate whether regeneration leads to rejuvenation specifically of the germline, we compared expression of germline genes between 2‐month‐old animals and intact and regenerated animals at different ages, and observed higher expression of germline genes after regeneration at all ages, and no clustering of the older regenerated samples with the young samples in principal component analysis based on germline‐specific genes (Figure S1).

### 2.3 Age‐related changes in gene expression

**Figure 4:**
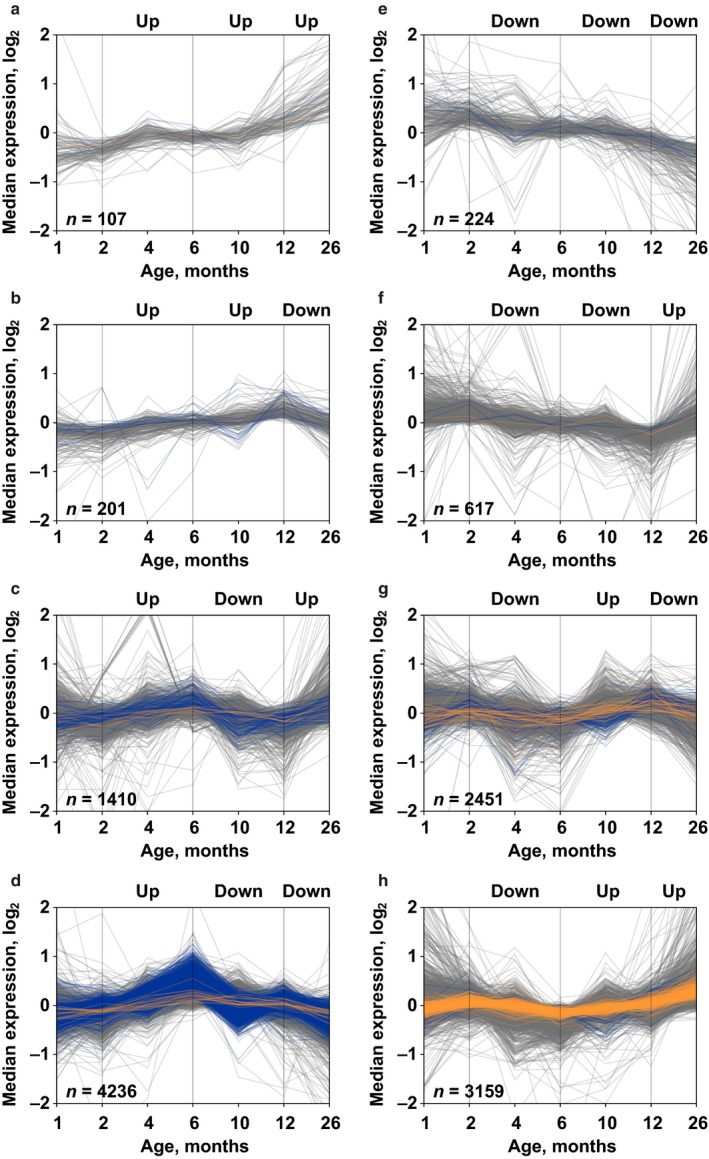
Temporal patterns of gene expression as a function of age. By dividing the studied period into three intervals (2–6 months, 6–12 months, and 12–26 months), eight temporal patterns of gene expression can be identified. Transcripts enriched in proliferating germline cells are represented in blue, and transcripts enriched in somatic neoblasts in orange

To study age‐related patterns and changes in gene expression, the different regenerative conditions were pooled to increase statistical power as we showed that the impact of single and multiple regenerations is limited. We used ebseq‐hmm software (Leng et al., 2015) to compute statistically significant time patterns. To reduce the complexity, the 4M and 10M samples were not included in this analysis. Based on the 2–6M, 6–12M, and 12–26M time intervals, eight patterns of changes in gene expression could be identified (Figure 4). The Up‐Down‐Down and Down‐Up‐Up patterns are the most abundant, with 4,236 and 3,159 genes, respectively (Figure 4d,h). Remarkably, the Up‐Down‐Down pattern had an 5.94‐fold enrichment for proliferating germline cell transcripts (*p* < 2.2e‐16), while the Down‐Up‐Up pattern had an 3.72‐fold enrichment (*p* < 2.2e‐16) for transcripts of the somatic neoblast list (Table 1), suggesting that the reproductive function in *M. lignano* is increasing from 2 to 6 months of age and then declines from 6 months onwards, whereas neoblast activity is lower in mature 6‐month‐old animals compared to young adults, but increases in the later life of the animals.

**Table 1:** Enrichment of various gene categories among genes differentially expressed with age

Dataset	Expression with age	GenAge	Genome maintenance	Germline	Neoblasts
*Macrostomum lignano* a	Down‐Down‐Down	ns	ns	ns	ns
	Down‐Down‐Up	ns	ns	0.13	0.15
	Down‐Up‐Down	ns	ns	0.49	ns
	Down‐Up‐Up	1.38	1.51	0.25	3.72
	Up‐Down‐Down	ns	ns	5.94	0.24
	Up‐Down‐Up	ns	ns	ns	0.3
	Up‐Up‐Down	ns	ns	ns	ns
	Up‐Up‐Up	ns	ns	ns	ns
	Increased at 26M vs. 2M	1.36	ns	0.58	2.93
	Decreased at 26M vs. 2M	0.75	0.46	1.49	0.07
*C. elegans* b	Increased at D10 vs. D3	0.61	0.45		
	Decreased at D10 vs. D3	ns	ns		
Mouse intestinal	Increased at 20M vs. 2M	ns	0.23		
Stem cells^c^	Decreased at 20M vs. 2M	ns	ns		
Mouse liver^d^	Increased at 21M vs. 3M	ns	0.41		
	Decreased at 21M vs. 3M	ns	ns		
Mouse skin^e^	Increased at 30M vs. 2M	ns	0.39		
	Decreased at 30M vs. 2M	0.74	ns		
Zebrafish skin^e^	Increased at 42M vs. 5M	0.66	0.41		
	Decreased at 42M vs. 5M	ns	1.47		

ns, not significant.

a This study.

b Rangaraju et al., 2015.

c Nalapareddy et al., 2017.

d Bochkis et al., 2014.

e Mansfeld et al., 2015.

Next, we classified *M. lignano* transcripts according to their homology to human genes present in the GenAge database of aging‐related genes (De Magalhães & Toussaint, 2004) and to the genome maintenance genes (Macrae et al., 2015), and overlapped the distribution of these gene categories with the identified temporal groups. Remarkably, the Down‐Up‐Up category is 1.38‐fold enriched (*p* = 4.293e‐08) for aging‐related genes, and 1.51‐fold enriched (*p* = 6.961e‐05) for genome maintenance genes (Table 1).

When comparing genes differentially expressed between 2‐month‐old and 26‐month‐old animals, the enrichment for the GenAge category among genes with elevated expression remains (1.36‐fold enrichment, *p* = 4.625e‐12), while this category is significantly underrepresented among genes with decreased expression (0.75‐fold depletion, *p* = 2.066e‐06; Table 1). At the same time, the genome maintenance category is not enriched in genes upregulated between 2‐month‐old and 26‐month‐old animals but is significantly underrepresented among downregulated genes (0.46‐fold depletion, *p* = 4.541e‐08; Table 1).

To evaluate whether the observed enrichments for GenAge and genome maintenance genes among genes differentially expressed with age in *M. lignano* are also typical for conventional model organisms, we re‐analyzed several publicly available aging RNA‐seq datasets, including *C. elegans* (Rangaraju et al., 2015)*,* mouse intestinal stem cells (Nalapareddy et al., 2017) and liver (Bochkis, Przybylski, Chen & Regev, 2014), and mouse and zebrafish skin (Mansfeld et al., 2015). In contrast to *M. lignano*, none of these datasets show enrichment for GenAge genes among genes upregulated with age, and in *C. elegans* and zebrafish skin, this category is even significantly underrepresented (Table 1). Similarly, the depletion of GenAge genes observed in genes downregulated with age in *M. lignano* is not present in other models, except mouse skin (Table 1). Furthermore, in contrast to *M. lignano,* genome maintenance genes are significantly underrepresented among genes upregulated with age in all models, and even enriched among genes downregulated with age in zebrafish skin (Table 1).

Taken together, these findings indicate that gene expression undergoes temporal changes in *M. lignano*, which are at least partly due to aging, but the aging profile is very different from conventional models, such as *C. elegans,* mouse, and zebrafish.

### 2.4 Increased expression of “biological quality” genes is associated with old age in *M. lignano*

**Figure 5:**
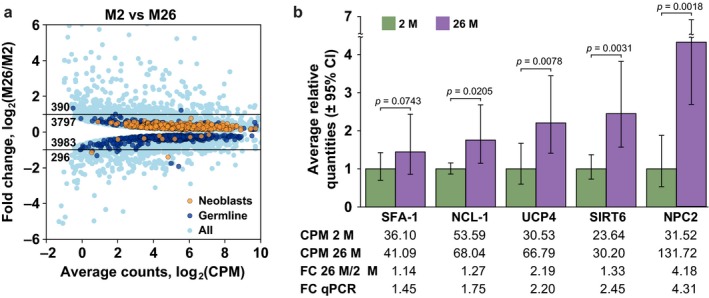
Differentially expressed genes between 2‐M‐old and 26‐M‐old animals. (a) Fold change plot of statistically significant differentially expressed genes, with neoblast and germline genes indicated in different color. (b) Verification of gene expression changes for selected genes by RT‐qPCR. The graph is normalized on the expression level of the target gene in the 2‐month‐old animals. The corresponding RNA‐seq data in the form of counts per million (CPM), with calculated fold changes, are given below the graph

The fact that genes from the genome maintenance, neoblast, and aging‐related categories are significantly enriched in the Down‐Up‐Up temporal gene expression path and between young and old animals (Table 1), combined with the finding that *M. lignano* animals can live multiple years and can maintain their regenerative capacity with advancing age (Figure 1b), lead us to the hypothesis that genes that can attenuate and offset consequences of aging are actively regulated in *M. lignano*. To further explore this hypothesis, we focused on the two extremes available in our dataset and compared gene expression changes between 2‐month‐old and 26‐month‐old worms. The comparison revealed 4,187 significantly upregulated and 4,279 significantly downregulated genes (Figure 5a). The downregulated transcripts represent a GO term enrichment related to ion transport and the plasma membrane. Furthermore, the cytoskeletal part and cilium components are enriched (Table S4). Interestingly, germline genes are overrepresented in the downregulated gene set (1.49‐fold enrichment, *p* < 2.2e‐16), while genes enriched in somatic neoblasts are underrepresented (0.07‐fold enrichment, *p* < 2.2e‐16) (Table 1). The GO term analysis of genes significantly upregulated at old age shows a clear enrichment for the ribosomal subunit and translation, but also for the extracellular region component (Table S4). The upregulated genes are enriched for the age‐related genes (1.36‐fold enrichment, *p* = 4.625e‐12) and for somatic neoblast transcripts (2.93‐fold enrichment, *p* < 2.2e‐16) (Table 1). Interestingly, the majority of genes known to be essential for neoblast functionality during cell renewal and regeneration in *M. lignano* have an increased expression with old age: PIWIL1, DDX39B, CDK1, RRM1, H2AFV (Table S5). As a knockdown of these genes results in phenotypes related to neoblast malfunctioning, an increasing expression at old age suggests that *M. lignano* invests in maintaining stem cell functionality with advancing age.

A detailed analysis of enriched GO term categories among genes upregulated with age revealed a particularly interesting term “Regulation of biological quality” (GO:0065008), containing 373 human homolog genes, of which 28 are also in the GenAge database of aging‐related genes (Table 2). This list contains genes known to have positive influence on longevity, such as SIRT6 (Kanfi et al., 2012), PRMT1 (Takahashi et al., 2011), or PCK1 (Hakimi et al., 2007). Remarkably, however, the positive effect of these genes on longevity in the studied models is seen particularly upon overexpression of these genes. In contrast, it appears that in *M. lignano,* such genes are naturally upregulated with age. We hypothesize that such genes are, to large extent, responsible for the prolonged longevity observed in *M. lignano*.

**Table 2:** “Regulation of Biological Quality” homologs upregulated with age in *Macrostomum lignano*

AACS, AAK1, ABCB1, ABCG2, ACACA, ACADVL, ACE, ACHE, ACO1, ACP5, ACTB, ACTG1, ADCY1, ADD1, ADIPOR2, AK3, AKAP1, AKAP10, AKR1B1, ALDH9A1, AMPD2, ANK2, ANK3, ANPEP, **APEX1,** APOB, APRT, AQP9, ARF1, ARF6, ARFGEF1, ARID2, ARL2, ARPC2, ARRDC3, ASGR2, **ATM,** ATP2A1, ATP2A2, ATP2C1, ATP5B, ATP6V1D, ATP6V1E1, ATP6V1G1, BAG3, BCAP31, BCAT2, BEST2, BOLL, **BRCA2,** C10orf112, C19orf12, CA12, CA7, CACNA1D, CACNB2, CAD, CALR, CAPN3, CAPZB, CARHSP1, CASP3, CAV1, CAV3, CBX5, CCT2, CCT3, CCT4, CCT6A, CCT7, CCT8, CD81, CDC20, **CDC42,** CDK5RAP1, **CDK7,** CDO1, CEL, CFL1, CHRNA10, CHRNA2, CHRNA4, CLNS1A, CNNM2, COPA, CORO1B, CTSA, CUBN, CYP17A1, DCLRE1A, DHCR7, DKC1, DLG1, DLL1, DMPK, DNA2, DNLZ, DSP, DYNLL2, ECE2, EIF4A3, EIF4EBP2, ERBB4, EXOSC10, EXOSC2, EXOSC3, EXOSC6, EXOSC9, F5, FABP3, FAM46A, FBN1, FGA, FLOT1, FLOT2, FMNL1, FN1, FTH1, G3BP2, GAA, GAPDH, GATA4, GCKR, GLRX3, GLRX5, GLUD1, GLUL, GM2A, GNAS, GNB2L1, GNL3L, GOLGA7, GPI, GRID1, GRIK1, GRIK2, GRIN3B, GRM5, **GSK3B,** GSTO1, H3F3B, HCK, HIST2H4A, HK1, **HMGB1, HMGB2,** HNRNPA1, HNRNPA2B1, HNRNPC, HNRNPU, HSD17B12, HSD17B14, HSD17B4, HSD17B6, HSD17B8, **HSP90AA1,** HSP90B1, HSPA5, **HSPD1,** HYPK, IARS, ICA1, IDE, IGF2BP1, **IRS1,** ITPR1, KCNH7, KCNK16, KCNN2, KDELR2, KIAA1715, LCK, LPCAT1, MAOA, MAP3K4, MEN1, METTL14, MEX3D, MOXD1, MUC2, MUC4, **MXI1,** MYH10, MYH6, MYH7, MYL12B, MYLIP, MYRIP, NAALAD2, NANOS1, NAPA, **NBN,** NCLN, NEK2, NF1, NLE1, NLGN4X, NLK, NOX3, NPC1, NPC2, NPR2, NR2F2, NXN, P2RX2, P4HB, PABPC1, PAFAH2, PAH, PBLD, PCID2, **PCK1,** PCK2, PDIA3, PDIA6, PHB, PHB2, PHPT1, PIF1, PKD1, PKD2, PLB1, PLK2, PLSCR1, **POLA1, POLG,** POSTN, PPA2, **PPARG,** PPIB, PPIF, PPP2R3C, PPT1, PRDX4, PRDX6, PRELID1, PRKAA2, PRKAR2A, PRMT1, PROC, PSMA1, PSMA3, PSMA5, PSMB3, PSMB4, PSMB6, PSMB7, PSMD10, PSMD4, PTGES2, PTGES3, **PTGS2, PTPN11,** PTPRC, PUM2, QRSL1, RAB11A, RAB1A, RAC1, RAD23A, RAD51C, RDH13, RDX, RETSAT, RFC3, RFC4, **RGN,** RGS14, RHAG, RHOA, **RPA1,** RPA2, RPA3, RPL11, RPL23, RPL5, RPS17, RPS24, RPS27A, RPS5, RPS6, RPS7, RPS9, SAA1, SCARB1, SEL1L, SERBP1, SET, SEZ6, SGPL1, SH3GLB1, SHANK3, **SIRT6,** SLC1A3, SLC1A6, SLC25A5, SLC26A2, SLC26A5, SLC30A1, SLC30A2, SLC30A7, SLC31A1, SLC34A1, SLC34A2, SLC39A14, SLC46A1, SLC4A8, SLC8A1, SLC8B1, SLC9A6, SLC9A8, SLMAP, SMAD2, SMAP1, **SOD1,** SPTBN1, **SQSTM1,** SRC, SREBF1, STAR, **STAT5A,** STIM1, STX1A, STXBP5, STXBP5L, SYNCRIP, SYT7, SYTL4, TAF9, TARDBP, TCP1, TFPI, TMEM165, TMEM97, TMOD3, TMX1, TNKS2, TNNT2, TOMM7, TPM1, TRA2B, TRIAP1, TRIM71, TRIOBP, TRPA1, TRPM1, TRPM2, TRPM8, TRPV5, UBA52, UMPS, UNC13B, VAMP2, VAMP8, VAPB, VARS, VAV1, VIL1, VWF, WFS1, **WRN,** XBP1, XRCC1, **XRCC5,** YBX1, YBX3, YTHDF2, YWHAB, YWHAE, **YWHAZ,** ZFP36, ZFP36L1, ZNF236

Genes from the GenAge database are in bold.

To independently validate gene expression changes observed in our RNA‐seq data, we selected several genes with known effects on lifespan in other models, and which appear to be upregulated with age in *M. lignano*. Specifically, we focused on homologs of SFA‐1, NCL‐1, and UCP2/4 genes, which extend lifespan in *C. elegans* upon overexpression (Heintz et al., 2016; Sagi & Kim, 2012; Tiku et al., 2017), and SIRT6 gene, which extends lifespan in male mice upon overexpression (Kanfi et al., 2012). We also selected gene NPC2 as a positive control for RT‐qPCR, as it is among the highest age‐upregulated genes according to the RNA‐seq data (Table S5). Three cohorts of 2‐month‐old and three cohorts of 26‐month‐old animals were used for the quantification of expression of the selected genes by RT‐qPCR. In all cases, RT‐qPCR confirmed fold changes inferred from RNA‐seq data with good correlation and statistical significance (Figure 5b).

## 3 DISCUSSION

### 3.1 Regeneration does not affect aging of *M. lignano*

One of the main aims of this study was to investigate whether the sexual flatworm *M. lignano* can rejuvenate by regenerating its body. All studied characteristics (mortality rate, fertility, morphology, and gene expression) indicated that this is not the case. While the mortality rate, morphology, and gene expression data are unambiguously supporting this conclusion, the fertility data are more difficult to interpret. At the age of 12 months, multiple cut worms have significantly lower fertility than intact worms, but at 26 months, multiple cut worms have a significantly increased fertility compared to intact worms. The most likely explanation for the restored fertility at 26 month after multiple cutting is the selection imposed on the animals due to the multiple cutting. We hypothesize that repeated amputation of the body could represent a condition with an increased selection for more fit worms. It is possible that some of the unfit worms died between 300 and 500 days in the multiple cut condition, leading to an increased fraction of the fitter worms in the survived population. As a result, when animals were randomly selected for pairing in the fertility experiment at 26 months, more fertile couples were formed than at 12 months.

All data taken together indicate that regeneration does not cause rejuvenation in *M. lignano*. Moreover, the gene expression data demonstrate the dominant effect of age over single or multiple regeneration. This is in contrast with the previous observations (Egger et al., 2006) showing lifespan extension due to repeated regeneration. The maximum lifespan of 10 months of the uncut control group of that experiment is, however, remarkably short compared to the later published (Mouton et al., 2009) and here obtained lifespans showing that worms are able to live over 2 years. The difference suggests non‐age‐related causes of death in the controls of the original experiment. The frequent presence of Thraustochytrids in laboratory cultures before the description of its pathogenic effect on *Macrostomum* (Schärer, Knoflach, Vizoso, Rieger & Peintner, 2007) might be such a cause. These unicellular organisms are known to cause degradation of worms starting at the caudal end. Therefore, repeated amputation could have resulted in the removal of Thraustochytrids and as a consequence decreased mortality in regenerating animals compared to noncut controls.

### 3.2 *M. lignano* can offset negative consequences of aging

The lack of rejuvenation in *M. lignano* is in line with the results observed in planarians (Tan et al., 2012), focusing on telomere shortening and elongation in *Schmidtea mediterranea*. Telomere length and telomerase activity indicated that regeneration can cause a reversal of aging in the asexual, but not the sexual strain. Combined with our data, this suggests that rejuvenation might be limited to asexual flatworms for which regeneration is an integral part of the reproduction. In sexual species, uncoupling of regeneration from reproduction might have led to a loss of the rejuvenating abilities. One should, however, keep in mind that even in sexual flatworms, there is no strict separation between germline and soma, as amputated heads can regenerate the gonads (Egger et al., 2006). Maybe not surprising, we found an overrepresentation of transcripts enriched in proliferating germline cells within the small group of transcripts with a long‐lasting change in expression after regeneration. How the youthfulness of the germline is ensured remains a question. A hypothesis helping to explain this is the presence of a highly conserved population of primordial stem cells (PriSCs) within the metazoa. These cells are characterized by a mixed germ/soma potential, but fall within the germline definition. The abundance of PriSCs is suggested to depend on the reproductive strategy and might be restricted or even rudimentary in sexual animals (Solana, 2013).

Despite the lack of rejuvenating capabilities, *M. lignano* shows a fascinating aging pattern. Firstly, worms have a long lifespan of several years as 69 ± 4% of the nontreated worms are still alive after 26 months. This is surprisingly longer than the previous demographic study of *M. lignano*, where the 90th percentile lifespan was reached after about 1 year (Mouton et al., 2009). Several differences in experimental setup might, at least partly, explain this. While in the previous study worms were maintained individually in 12‐well plates and transferred every 4 weeks, in the current study, worms were maintained in groups starting with 100 worms in Petri dishes with a diameter of 90 mm and transferred twice a week. Consequently, there are differences in reproductive behavior and food quality. In addition, the previous study used a wild‐type strain which is not available anymore. The current study was performed with the inbred DV1 line, which was used for establishing the genome and transcriptome assemblies (Grudniewska et al., 2016; Wudarski et al., 2017). During the current survival studies, it was, however, shown that the DV1 line has a chromosome duplication, which has unknown effects on the biology of the worms (Wudarski et al., 2017; Zadesenets, Ershov, Berezikov & Rubtsov, 2017). Another fascinating observation is that *M. lignano* does not show signs of a decreased regeneration capacity with advancing age, even after up to twelve amputations of the body, which is in strong contrast to mammals (Sharpless & DePinho, 2007). This corresponds to Egger's observations during the multiple regeneration studies with the wild‐type strain, as he also indicated that the regeneration rate did not decline with advancing age and increasing number of amputations (Egger et al., 2006). *M. lignano* is not the only animal which can maintain its regenerative capacity with increasing age. Examples are planaria (Tan et al., 2012), *Hydra* (Hobmayer et al., 2012), and sea urchins (Bodnar & Coffman, 2016), but also regeneration of the fins and heart in zebrafish (Itou, Kawakami, Burgoyne & Kawakami, 2012), and regeneration of the lens in newts (Eguchi et al., 2011).

### 3.3 Stem cells: Key players in strategies to attenuate aging

The lack of a clearly increasing mortality rate with advancing age and the age‐independent regeneration capacity of *M. lignano* reminds of *Hydra*, which is shown to be practically immortal (Martínez, 1998; Schaible et al., 2015). As *Hydra*, flatworms possess stem cells, including a pluripotent subpopulation (Hobmayer et al., 2012; Wagner, Wang & Reddien, 2014), which can replace damaged and old cells and therefore attenuate aging. In contrast to *Hydra*, where this system seems to be brought to perfection, signs of inefficiency at the tissue level are observed in *M. lignano*, as hallmarks of aging, such as the appearance of cysts and loss of eyes, are present. We observed that cysts could affect fitness on the organismal level. Based on their size, number, and location, they can limit for instance motility and feeding behavior, which in turn, might lead to the death of the individual. Despite this difference, *Hydra* and flatworms might share a common stem cell‐based strategy to slow down or avoid aging characteristics. The high cellular turnover during homeostasis seems to be the core of this strategy. It has been estimated that in *M. lignano*, about 500 of the total of about 25,000 cells are replaced on a daily basis (Ladurner et al., 2008). This constant replacement of aged and damaged cells by new cells formed by the neoblasts might represent a powerful strategy to maintain the body during aging.

The central role of stem cells in *M. lignano* longevity is further confirmed by the upregulation of the previously identified transcripts essential for neoblast functionality (Grudniewska et al., 2016) at old age, and the gene expression changes in genes related to cell cycle, such as RHAB, and related to the DNA damage response, such as SESN1 and SIRT6. This should not be surprising as a decrease in stem cell functionality is an integrative hallmark of aging (Lopez‐Otin, Blasco, Partridge, Serrano & Kroemer, 2013), while the maintenance of it is a cornerstone of naturally evolved mechanisms of fighting aging. Different invertebrate and vertebrate long‐lived species developed independent strategies for repressing aging, but often their life trajectory is related to cell cycle control, telomere and genome maintenance, and tumor suppression (Gorbunova, Seluanov, Zhang, Gladyshev & Vijg, 2014; Ma et al., 2016).

### 3.4 Temporal gene expression changes in *M. lignano* as a resource for aging research

While a large number of genes were found to be differentially expressed in old worms compared to young ones, 4,187 up and 4,279 down, the fold change of these age‐related changes in gene expression was rather small. Only 390 of the significantly upregulated and only 296 of the significantly downregulated genes had a log fold change larger than one. This should, however, not be a concern as the literature already stated that gene expression changes with aging tend to be subtle compared to changes in other research fields using RNA‐Seq such as characterizing the causes of specific diseases or cancer (Gribble & Mark Welch, 2017). Often research focusses on experimental manipulation of gene expression, causing large expression changes, to study the role of a specific gene in aging and lifespan. Focusing on the subtle genome wide changes in expression, however, provides an ideal baseline for evaluating expression changes due to interventions and even to develop new hypotheses for the molecular basis of aging (Gribble & Mark Welch, 2017). To improve our understanding of the transcriptional regulation of aging, it is important to compare expression profiles of different animals, and each dataset is an important step for establishing the aging transcriptome. Meta‐profiling will show which age‐related changes in expression are conserved and which ones species‐specific. While the first is interesting for the development of biomarkers and understanding fundamental aging processes, the latter might reveal interesting novel therapeutic approaches to improve human healthspan and facilitates engineering approaches to healthspan extension (Sagi & Kim, 2012). For this purpose, it will be interesting to follow up on the longevity genes which are upregulated with age in *M*. *lignano*. To facilitate the use of our data in future research, we annotated the homology of *Macrostomum* transcripts to both human and *C. elegans* genes (Table S4) and developed a user‐friendly web interface to access and query the data (http://ageing.macgenome.org/).

## 4 CONCLUSIONS

We showed that the flatworm rejuvenation theory is not valid in the sexual species *M. lignano*. However, all data taken together reveal that the regulation of fundamentally conserved mechanisms of cellular and organismal health maintenance evolved in *M. lignano* to efficiently offset negative consequences of aging. Therefore, we advocate that *M. lignano* is a novel powerful model toward studies of aging‐related genetic pathways, and we provide temporal gene expression patterns of aging in *M. lignano* as a resource to tap the power of this model.

## 5 EXPERIMENTAL PROCEDURES

### 5.1 Experimental model

The inbred line of the hermaphroditic free‐living flatworm species *Macrostomum lignano*, called DV1, was cultured in Petri dishes with nutrient‐enriched artificial seawater (f/2). Worms were kept at 20°C with a 14 hr/10 hr light/dark cycle and were fed ad libitum with the diatom *Nitzschia curvilineata*.

### 5.2 Aging and survival cultures of *Macrostomum lignano*

The aging culture consisted out of cohorts of 100 worms per plate which were transferred every 3 or 4 days, to ensure age synchronicity as eggs hatch after about 5 days. The aging experiment included three different conditions: intact (NC), once cut (OC), and multiple cut (MC) worms. Intact individuals were following the general culturing procedures, without any additional manipulations. The once cut condition refers to worms which were cut in the region between the pharynx and gonads 2 months prior collection. The head was left to regenerate while the amputated body part was discarded. Finally, multiple cut worms underwent this amputation procedure several times with 2‐month intervals between successive amputations. The last amputation was performed 2 months before collection. Intervals of 2 months were chosen to ensure complete regeneration between amputations and before collection as heads can regenerate into functional adults within about 3 weeks (Egger et al., 2006). The survival of the MC and NC condition was studied. For both conditions, five replicate cohorts, each starting with 100 individuals, were followed.

### 5.3 Morphology as a function of age

Morphological changes as a function of age were studied at the 2M, 12M, and 26M time points for NC, OC, and MC worms. In addition, for each condition at each time point, three replicates of 30 individuals were randomly selected to quantify the percentage of animals displaying cysts. For both experiments, animals were carefully observed under a stereoscope. To obtain photographs, animals were washed in f/2 medium, relaxed in 1:1 f/2:MgCl_2_ (7,14%), and put in a small drop in a Petri dish. Pictures were made using EVOS XL Core Imaging System (ThermoFisher).

### 5.4 Fertility as a function of age

The fertility of NC, OC, and MC worms was determined at the age of 12 and 26 months. To study the effect of age on fertility, intact 2M worms were used as young controls for both time points. For the regenerative conditions, worms were amputated for the last time 2 months before the fertility experiment to ensure complete regeneration. One week before the experiment, 20 individuals were randomly selected from the aging cultures and maintained alone to lay all developing eggs. Afterward they were randomly divided into 10 pairs of worms and maintained as couples for 4 weeks. Individuals and couples were cultured in small Petri dishes (35 mm diameter) with f/2 medium and diatoms. They were weekly transferred to new Petri dishes to ensure ad libitum food sources. The old dishes were kept, and the number of hatched worms was quantified as a measure for fertility.

### 5.5 Preparation and sequencing of RNA‐seq libraries

Worms of the different conditions were collected at several ages for RNA sequencing. At the 26M, 12M, 10M, 8M, and 6M ages, NC, OC, and MC worms were collected. At the 4M time point, both OC and NC worms were collected. At the 2M and 1M time points, NC worms were collected (Figure 1a). For each, three replicates of 20 individuals were rinsed with fresh f/2 medium, suspended in 500 μl of TRIzol reagent (Ambion), and stored at −80°C.

Total RNA was extracted from the samples with the Direct‐zol RNA MiniPrep Kit (Zymo Research), following the manufacturer's protocol. The concentration of the extracted RNA was assessed using the Qubit RNA BR Assay Kit (Life Technologies), and RNA‐Seq libraries were generated with the SureSelect Strand‐Specific RNA Library Prep Kit for Illumina multiplexed sequencing (Agilent) in accordance with the manufacturer's instructions. Libraries were pooled, 16 samples per run (2 nm), and sequenced on the Illumina HiSeq 2500 machine.

### 5.6 Differential expression analysis of RNA‐Seq data

Illumina sequencing reads were mapped to the annotated Mlig_3_7 genome assembly (Wudarski et al., 2017) using star v.2.5.2b (Dobin et al., 2013) in the transcriptome quantification mode with following parameters “–outFilterMultimapNmax 30 –outFilterMismatchNmax 5 –quantMode TranscriptomeSAM.” To generate read counts for transcript clusters, the resulting bam files were processed by Corset v.1.06 (Davidson & Oshlack, 2014). EdgeR package and EBSeq‐HMM (Leng et al., 2015) were used to perform differential gene expression analysis.

A user‐friendly web interface was made to facilitate the access and query of the age‐related gene expression dataset of *Macrostomum lignano*: http://ageing.macgenome.org.

For the analysis of gene set enrichment in conventional model organisms during aging, we retrieved from NCBI Short Read archive RNA‐seq for *C. elegans* (Rangaraju et al., 2015), mouse intestinal stem cells (Nalapareddy et al., 2017), mouse liver (Bochkis et al., 2014), and mouse and zebrafish skin (Mansfeld et al., 2015). Annotated genomes were retrieved from Ensembl, and RNA‐seq data were mapped and quantified using star v.2.5.2b (Dobin et al., 2013).

### 5.7 RT‐qPCR validation of differentially expressed genes

To confirm the age‐related upregulation of genes with positive longevity effects, we performed RT‐qPCR on a subset of them. The selection of genes and their primers are listed in Table S6. The primers of the target and reference genes were designed with IDT's online PrimerQuest Tool, using the Mlig_3_7 assembly of the genome and transcriptome (Wudarski et al., 2017) and requiring that at least one primer in each pair spanned exon–exon splice junction. Three replicates of 2‐month‐old and three replicates of 26‐month‐old animals were used for RNA extraction. Each replicate consisted of 20 individuals, which were rinsed with fresh f/2 medium, suspended in 500 μl of TRIzol reagent (Ambion), and stored in −80°C until use. Total RNA was extracted from the samples with the Direct‐zol RNA MiniPrep Kit (Zymo Research), following the manufacturer's protocol. RNA concentrations were measured with the NanoDrop 2000 spectrophotometer (Thermo Scientific) and adjusted to the same concentration. The RNA was reverse transcribed to generate cDNA using SuperScript IV Reverse Transcriptase (Thermo Fisher Scientific), and a mix of oligo(dT) and Random Hexamer primers. qPCR was performed with the iTaqTM Universal SYBR Green Supermix (Bio‐Rad) using the Light Cycler 480 system (Roche Life Sciences). Primer efficiency was calculated within Light Cycler 480 software and is based on a twofold dilution series. Gene expression analysis and statistics were performed with qbase+ software.

### 5.8 Statistical analysis

For the nonmolecular experiments, ANOVA with post hoc Bonferroni tests was used for normally distributed data, or Kruskal–Wallis with post hoc Dunn's multiple comparison tests for not normally distributed data. A significance level of *p* < .05 was used. The specific choice of tests for experiments is reported in the Results section.

### 5.9 Accession numbers

RNA sequencing data were deposited to NCBI Short Read Archive under Accession no. SRR5876257‐SRR5876310.

## AUTHOR CONTRIBUTIONS

MG, SM, VG, and EB conceptualized and designed the study; acquired, analyzed, and interpreted the data; and drafted the manuscript. LG acquired and analyzed the data.

## CONFLICT OF INTEREST

None declared.
