# Supplementary figures and images for: Resilience to aging in the regeneration‐capable flatworm Macrostomum lignano
Source: Aging Cell. 2018 Feb 28;17(3):e12739. doi: 10.1111/acel.12739 (PMC5946080; doi:10.1111/acel.12739)

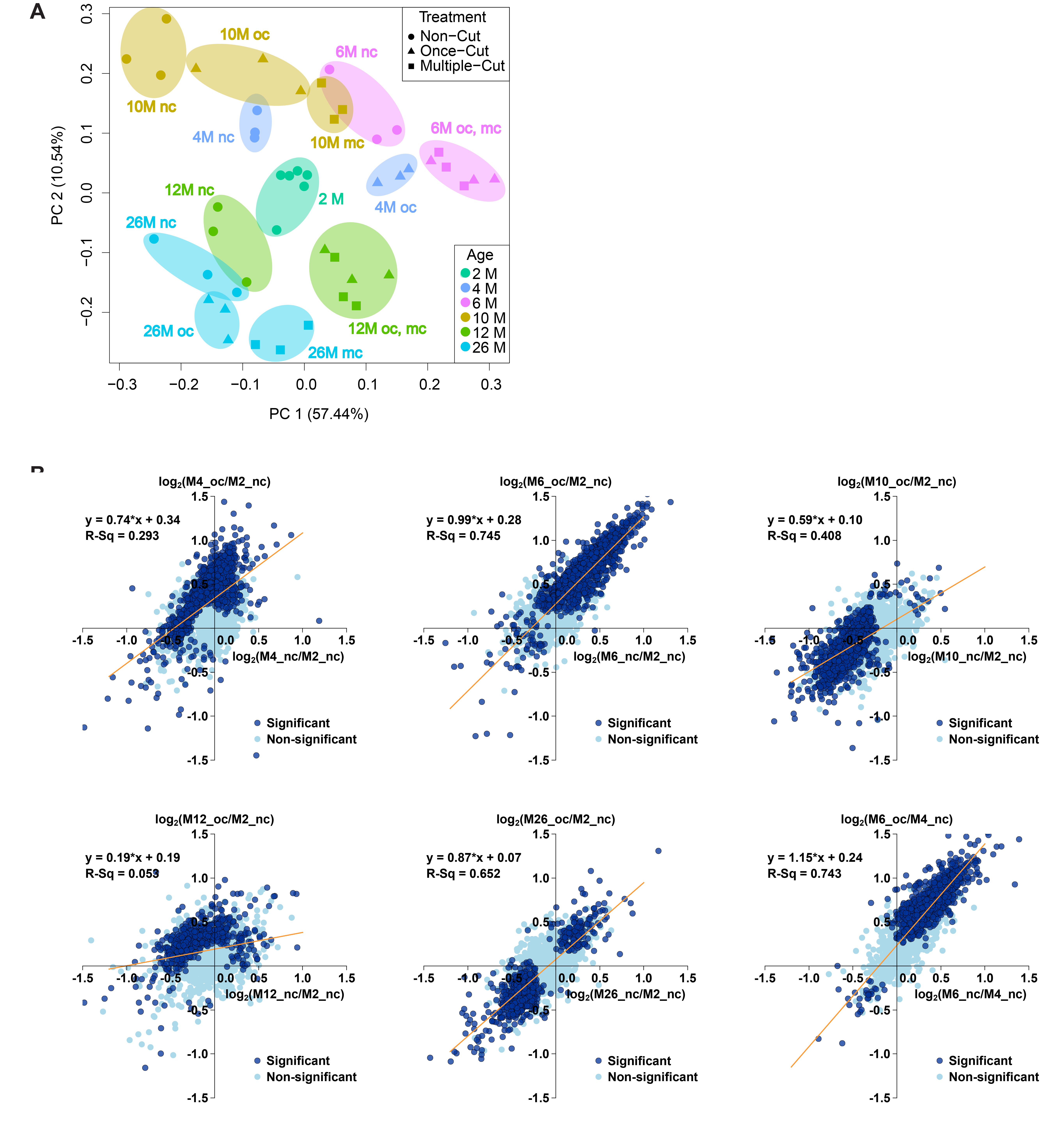

Supplement: Supplementary file 1 [file ACEL-17-e12739-s001.png]
