# Supplementary material for: Resilience to aging in the regeneration‐capable flatworm Macrostomum lignano
Source: Aging Cell. 2018 Feb 28;17(3):e12739. doi: 10.1111/acel.12739 (PMC5946080; doi:10.1111/acel.12739)
Supplement: Supplementary file 2 [file ACEL-17-e12739-s002.odt]

| Table S1 RNA-seq libraries generated in the study. |
| --- |
| library | age | treatment | culture | reads | accession |
| M1_nc_F_2 | 1M | nc | F | 16,717,817 | SRR5876294 |
| M1_nc_F_3 | 1M | nc | F | 20,241,961 | SRR5876293 |
| M1_nc_F_4 | 1M | nc | F | 7,543,105 | SRR5876296 |
| M1_nc_G_1 | 1M | nc | G | 12,718,885 | SRR5876295 |
| M1_nc_G_2 | 1M | nc | G | 16,692,275 | SRR5876298 |
| M1_nc_G_3 | 1M | nc | G | 16,338,706 | SRR5876297 |
| M2_nc_E_1 | 2M | nc | E | 16,846,625 | SRR5876300 |
| M2_nc_E_2 | 2M | nc | E | 9,787,771 | SRR5876299 |
| M2_nc_E_3 | 2M | nc | E | 12,746,303 | SRR5876292 |
| M2_nc_F_1 | 2M | nc | F | 18,156,235 | SRR5876291 |
| M2_nc_F_2 | 2M | nc | F | 16,293,663 | SRR5876260 |
| M2_nc_F_3 | 2M | nc | F | 14,020,942 | SRR5876259 |
| M4_nc_E_1 | 4M | nc | E | 19,190,321 | SRR5876258 |
| M4_nc_E_2 | 4M | nc | E | 27,997,746 | SRR5876257 |
| M4_nc_E_3 | 4M | nc | E | 23,143,665 | SRR5876264 |
| M4_oc_E_1 | 4M | oc | E | 13,115,498 | SRR5876263 |
| M4_oc_E_2 | 4M | oc | E | 22,300,935 | SRR5876262 |
| M4_oc_E_3 | 4M | oc | E | 21,893,283 | SRR5876261 |
| M6_nc_D_1 | 6M | nc | D | 19,190,837 | SRR5876266 |
| M6_nc_D_2 | 6M | nc | D | 17,635,425 | SRR5876265 |
| M6_nc_D_4 | 6M | nc | D | 6,514,190 | SRR5876275 |
| M6_oc_D_1 | 6M | oc | D | 17,931,625 | SRR5876276 |
| M6_oc_D_2 | 6M | oc | D | 18,413,566 | SRR5876273 |
| M6_oc_D_3 | 6M | oc | D | 16,218,046 | SRR5876274 |
| M6_mc_D_1 | 6M | mc | D | 15,421,413 | SRR5876279 |
| M6_mc_D_2 | 6M | mc | D | 17,119,732 | SRR5876280 |
| M6_mc_D_4 | 6M | mc | D | 8,969,833 | SRR5876277 |
| M10_nc_A_1 | 10M | nc | A | 17,704,719 | SRR5876278 |
| M10_nc_A_2 | 10M | nc | A | 16,076,433 | SRR5876271 |
| M10_nc_A_3 | 10M | nc | A | 13,160,069 | SRR5876272 |
| M10_oc_A_2 | 10M | oc | A | 12,816,510 | SRR5876304 |
| M10_oc_A_3 | 10M | oc | A | 14,203,347 | SRR5876303 |
| M10_oc_A_4 | 10M | oc | A | 12,114,601 | SRR5876306 |
| M10_mc_A_1 | 10M | mc | A | 17,257,794 | SRR5876305 |
| M10_mc_A_2 | 10M | mc | A | 13,670,424 | SRR5876308 |
| M10_mc_A_3 | 10M | mc | A | 11,738,596 | SRR5876307 |
| M12_nc_A_1 | 12M | nc | A | 7,845,963 | SRR5876310 |
| M12_nc_A_2 | 12M | nc | A | 11,997,093 | SRR5876309 |
| M12_nc_A_3 | 12M | nc | A | 9,979,059 | SRR5876302 |
| M12_oc_A_1 | 12M | oc | A | 9,856,656 | SRR5876301 |
| M12_oc_A_2 | 12M | oc | A | 13,032,688 | SRR5876283 |
| M12_oc_A_3 | 12M | oc | A | 10,718,426 | SRR5876284 |
| M12_mc_A_1 | 12M | mc | A | 14,706,581 | SRR5876285 |
| M12_mc_A_2 | 12M | mc | A | 10,572,149 | SRR5876286 |
| M12_mc_A_3 | 12M | mc | A | 10,546,447 | SRR5876287 |
| M26_nc_H_1 | 26M | nc | H | 18,348,977 | SRR5876288 |
| M26_nc_H_2 | 26M | nc | H | 16,135,766 | SRR5876289 |
| M26_nc_H_3 | 26M | nc | H | 22,417,556 | SRR5876290 |
| M26_oc_H_1 | 26M | oc | H | 10,795,606 | SRR5876281 |
| M26_oc_H_2 | 26M | oc | H | 17,037,269 | SRR5876282 |
| M26_oc_H_3 | 26M | oc | H | 17,116,273 | SRR5876270 |
| M26_mc_H_1 | 26M | mc | H | 19,814,344 | SRR5876269 |
| M26_mc_H_2 | 26M | mc | H | 20,205,867 | SRR5876268 |
| M26_mc_H_3 | 26M | mc | H | 20,598,586 | SRR5876267 |
