# Supplementary material for: Resilience to aging in the regeneration‐capable flatworm Macrostomum lignano
Source: Aging Cell. 2018 Feb 28;17(3):e12739. doi: 10.1111/acel.12739 (PMC5946080; doi:10.1111/acel.12739)
Supplement: Supplementary file 3 [file ACEL-17-e12739-s004.odt]

| Table S3  Enrichment of various gene categories among differentially expressed genes. |
| --- |
| Comparison | Germline | Neoblast | GenAge | Genome Maintenance |
| M4_nc_vs_M4_oc.up | 7.99 | 0.05 | 0.56 | ns |
| M4_nc_vs_M4_oc.down | 0.18 | ns | ns | ns |
| M6_nc_vs_M6_mc.up | 9.26 | 0 | ns | ns |
| M6_nc_vs_M6_mc.down | 0 | ns | ns | ns |
| M6_nc_vs_M6_oc.up | 7.99 | 0 | 0.68 | ns |
| M6_nc_vs_M6_oc.down | 0 | 0.31 | ns | 0.26 |
| M6_oc_vs_M6_mc.up | 0.11 | ns | ns | ns |
| M6_oc_vs_M6_mc.down | ns | ns | ns | ns |
| M10_nc_vs_M10_mc.up | 9.33 | 0.08 | 0.61 | ns |
| M10_nc_vs_M10_mc.down | 0.10 | 0.06 | ns | 0.22 |
| M10_nc_vs_M10_oc.up | 10.41 | 0.13 | 0.56 | ns |
| M10_nc_vs_M10_oc.down | 0.13 | ns | ns | ns |
| M10_oc_vs_M10_mc.up | 8.93 | 0 | ns | ns |
| M10_oc_vs_M10_mc.down | ns | ns | ns | ns |
| M12_nc_vs_M12_mc.up | 8.58 | 0.41 | 0.62 | ns |
| M12_nc_vs_M12_mc.down | 0.10 | 0.23 | ns | ns |
| M12_nc_vs_M12_oc.up | 6.13 | 0.43 | ns | ns |
| M12_nc_vs_M12_oc.down | 0.22 | ns | ns | ns |
| M12_oc_vs_M12_mc.up | ns | ns | ns | ns |
| M12_oc_vs_M12_mc.down | ns | ns | ns | ns |
| M26_nc_vs_M26_mc.up | 5.59 | 2.19 | ns | ns |
| M26_nc_vs_M26_mc.down | 0.10 | 0.16 | ns | ns |
| M26_nc_vs_M26_oc.up | ns | ns | ns | ns |
| M26_nc_vs_M26_oc.down | 0.18 | ns | ns | ns |
| M26_oc_vs_M26_mc.up | 5.25 | ns | ns | ns |
| M26_oc_vs_M26_mc.down | ns | ns | ns | ns |
| ns – not significant |  |  |  |  |
