# Supplementary material for: Resilience to aging in the regeneration‐capable flatworm Macrostomum lignano
Source: Aging Cell. 2018 Feb 28;17(3):e12739. doi: 10.1111/acel.12739 (PMC5946080; doi:10.1111/acel.12739)
Supplement: Supplementary file 4 [file ACEL-17-e12739-s007.odt]

| Table S6 Primers used for qPCr validation of differentially expressed genes |
| --- |
| Mlig gene ID | Gene homolog | Primer ID | Primer sequence | Amplification efficiency |
| Mlig014936.g1 | COX5B | COX5B-Fwd COX5B-Rev | CACAGTCACTATCGTCAGGTTG CCGCAGTAGCACCTTTGATTA | 2.03 |
| Mlig033687.g1 Mlig033687.g2 Mlig033687.g3 | EIF1A | EIF1A-Fwd EIF1A-Fwd | CGATGCCGAAGAATAAAGGAAAG TCCTGACCATCCTCCTTGTA | 2.07 |
| Mlig029440.g2 Mlig029440.g4 Mlig029440.g6 | GAPDH | GAPDH-Fwd GAPDH-Rev | GGGCGTCAATGAGGATTCTT CGAATTCGTTGTTGATCACCTTAG | 2.09 |
| Mlig030930.g1 Mlig030930.g3 Mlig027909.g3 | NCL1 | NCL1-Fwd NCL1-Rev | AGTACCACATGAAATTCGGAGAG TTTGTGTCCGCCACGATTA | 1.84 |
| Mlig024849.g1 Mlig024849.g2 Mlig024849.g3 | NPC2 | NPC2-Fwd NPC2-Rev | AACACGATGAGCCCGAAAT CGGCGAGCGATGGATAAG | 2.06 |
| Mlig029819.g2 Mlig024025.g1 Mlig024025.g5 | SFA1 | SFA1-Fwd SFA1-Rev | GTACGTCACCTCGATGTCTTC GCTCGATGCCCTGCTTAATA | 2.19 |
| Mlig004036.g1 Mlig004036.g2 Mlig023098.g2 Mlig023098.g5 | SIRT6 | SIRT6-Fwd SIRT6-Rev | CTGCAACCTGCAGAATACG GAGTCATGAGCTCGTCTGT | 1.75 |
| Mlig005493.g1 Mlig005493.g2 Mlig005493.g3 | UCP4 | UCP4-Fwd UCP4-Rev | ACAAGATTATCGCGGCTTCA GAGAGGAACTGGACTGACTAGA | 2.08 |
